# Burr hole craniostomy versus minicraniotomy in chronic subdural hematoma: a comparative cohort study

**DOI:** 10.1007/s00701-021-04902-3

**Published:** 2021-07-30

**Authors:** Shaian Zolfaghari, Jiri Bartek, Isabelle Strom, Felix Djärf, San-San Wong, Nils Ståhl, Asgeir S. Jakola, Henrietta Nittby Redebrandt

**Affiliations:** 1 Department of Neurosurgery, Institution of Clinical Sciences, Lund University, Lund, Sweden; 2 Department of Neurosurgery, Karolinska University Hospital, Stockholm, Sweden; 3 Department of Neurosurgery, Rigshospitalet, Copenhagen, Denmark; 4 Department of Clinical Neuroscience and Medicine, Karolinska Institutet, Stockholm, Sweden; 5 Department of Neurosurgery, St. Olavs Hospital, Trondheim, Norway; 6 Department of Neurosurgery, Sahlgrenska University Hospital, Gothenburg, Sweden; 7 Department of Clinical Neuroscience, Institute of Neuroscience and Physiology, University of Gothenburg, Sahlgrenska Academy, Gothenburg, Sweden

**Keywords:** CSDH, Surgical method, Complications, Recurrence, Outcome

## Abstract

**Background:**

Chronic subdural hematoma (CSDH) is one of the most common neurosurgical diseases. In surgical management of CSDH, there is a lack of standardized guidelines concerning surgical techniques and a lack of consensus on which technique(s) are optimal. Neurosurgical centers have shown a wide variation in surgical techniques. The purpose of this study was to compare two different surgical techniques, one burr hole craniostomy with an active subgaleal drain (BHC) and minicraniotomy with a passive subdural drain (MC).

**Methods:**

We conducted a multicenter retrospective cohort study at two neurosurgical centers in Sweden which included patients with unilateral CSDHs that received surgical treatment with either BHC or MC. The primary outcomes in comparison of the techniques were 30-day mortality, recurrence rate, and complications according to the Landriel Ibañez grading system for complications.

**Results:**

A total of 1003 patients were included in this study. The BHC subgroup included 560 patients, and the MC subgroup included 443 patients. A 30-day mortality when comparing BHC (2.3%) and MC (2.7%) was similar (p = 0.701). Comparing recurrence rate for BHC (8.9%) and MC (10.8%) showed no significant difference (p = 0.336). We found that medical complications were significantly more common in the MC group (p = 0.001). Surgical complications (type IIb) was also associated with the MC group (n = 10, p = 0.003). Out of the 10 patients with type IIb complications in the MC group, 8 had postoperative acute subdural hematomas.

**Conclusions:**

BHC was comparable to MC concerning 30-day mortality rate and recurrence rates. We did, however, find that MC was significantly associated with medical complications and serious surgical postoperative complications.

## Introduction

Chronic subdural hematoma (CSDH) is one of the most common neurosurgical diseases with a reported incidence of 8 to 14 per 100,000 person-years [14]. The incidence is expected to rise significantly, projecting the surgical evacuation of CSDH to become the most common neurosurgical procedure by 2030 [3]. The disease primarily affects the elderly population, and people with risk factors such as trauma, antithrombotic medication, anticoagulants, and alcohol abuse have been identified [10, 11, 14]. The increasing incidence already has a socio-economic impact on current healthcare systems, which together with recurrence rates estimated around 10–20% and a non-negligible surgical morbidity adds to the complexity of CSDH management [1, 4, 14]. Optimizing surgical treatment can help in lowering morbidity and recurrence rates, as well as minimizing the socio-economic impact.

The most common surgical technique is considered to be the burr hole craniostomy (BHC) where 1–2 burr holes are drilled. In the setting of 2 burr holes, they are drilled 5–8 cm apart to allow for effective irrigation of the hematoma [22]. An alternative to this is the minicraniotomy (MC) where 2–3 burr holes are drilled in close vicinity and joined together into a larger bone defect followed by irrigation. Previous data has shown that BHC has the best cure-to-complication ratio [29]. Furthermore, data on BHC have not been able to prove a difference in outcome when comparing 1 and 2 burr holes when performing BHC; there is however limited data on outcomes when performing BHC with one burr hole under local anesthesia [5, 25].

It is considered the standard of care today to insert a postoperative drain to allow for further drainage of hematoma as it has shown to decrease mortality and reduce the risk of recurrence of CSDH [23]. Initial evidence was shown for passive subdural drains, but lately evidence has shown less risk of recurrence when using an active subgaleal drain compared to the passive subdural drain [24].

The aim of this study was to assess burr hole craniostomy with active subgaleal drain in comparison to minicraniotomy with passive subdural drain with an emphasis on recurrence rate, mortality, and risk of complications.

## Methods and materials

### Study cohort

The following multicenter retrospective comparative cohort study took place at Scania University Hospital and Karolinska University Hospital. All patients over the age of 18 diagnosed with a surgically evacuated CSDH were eligible for the study. The study had the following exclusion criteria: bilateral CSDHs, cerebral shunts, simultaneous intracranial hemorrhages, and patients with permanent residency outside of Sweden. The patients included from Scania University Hospital were treated between 2012 and 2016, while the patients included from Karolinska University Hospital were treated between 2006 and 2014. During the time periods, the surgical techniques and management of CSDH were not altered at either center. A total of 1003 patients were included in this retrospective cohort study.

### Surgical techniques

Patients were operated and managed with the following surgical techniques denoted as methods 1 and 2. Method 1 (i) was the standard surgical technique for evacuation of CSDH at Scania University Hospital. Method 2 (ii) was the standard surgical technique for evacuation of CSDH at Karolinska University Hospital. Surgeries were always to be conducted with local anesthesia unless special circumstances indicated the need of general anesthesia. In both centers, perioperative usage of antibiotics was administered prior to the initiation of surgery.

i. Two or three burr holes combined into a minicraniotomy. The hematoma was evacuated with irrigation after which a passive subdural drain was placed in the subdural space for 24 h, while the patients were immobilized in bed. The passive drain did not have any active suction but rather functioned by natural pressure gradients.
ii. Singular burr hole. The hematoma was evacuated with irrigation after which an active subgaleal drain was placed in the subgaleal space for 24 h, while the patients were immobilized in bed [24]. The active drain used active suction to drain the remaining fluids from the subdural space.

### Variables

The data from this study was retrieved using the electric medical journal systems at both hospitals and the respective radiological image databases for both hospitals. Baseline characteristics for this study included age, gender (male/female), Charlson comorbidity index (CCI) variables (index which predicts mortality based on weighted comorbidities) [18], preoperative Glasgow Coma Scale (GCS) score, antithrombotic medications, use of vitamin K antagonist (VKA), and radiological densities of the hematomas on computed tomography scans graded as the largest portion of the hematoma being hypodense, isodense, hyperdense, or with mixed densities. All patients underwent a CT of the head preoperatively. No postoperative head CTs were routinely performed unless clinically indicated.

Radiological data was retrieved in the form of midline shift (mm) and largest hematoma diameter in the axial plane (mm). Surgical data was retrieved in the form of the type of anesthesia used (local with sedation or general anesthesia), drainage system duration, recurrence rate (defined as new evacuation of CSDH on the same side within 3 months of the initial evacuation), and mortality at 30 days and 1 year past surgical date. Complications were registered according to Landriel Ibañez (classification system for complications after neurosurgical procedures) [15]. Complications were defined as any deviation from the normal postoperative course occurring within 30 days of surgery. Reoperation was not registered as a complication in our study.

### Statistical analysis

All statistical analyses were performed using IBM SPSS Statistics for Windows, version 25.0, Armonk, NY, IBM Corporation. Descriptive statistics including measures such as frequency (n), percentages, mean, and median were employed to further describe subgroup characteristics. Univariate (independent samples t-test and Mann–Whitney), Chi-squared, Fisher’s exact test, and Cox regression were performed to assess the endpoints of this study. Alpha level of significance was defined as P-value < *0.05* in all analyses. Adjusted residuals > 2 was considered to indicate a significance level < 0.05.

## Results

### Baseline characteristics

A total of 1003 patients were included in our study cohort from two neurosurgical centers. A summary of the baseline characteristics can be found in Table 1. The majority of the study cohort consisted of males (68.2%) and were evenly distributed between the two treatment groups. The mean age of the study population at the time of diagnosis was 75 years. The two treatment groups had a similar distribution of underlying comorbidities defined as CCI > 1 point (33.9% vs 28.7%, p = 0.075 Mann–Whitney).

**Table 1:** Baseline characteristics of the study population. Variables are stratified for the two surgical methods

Variable, No. (%)	BHC with active subgaleal drain N = 560 No. (%)	Minicraniotomy with passive subdural drain N = 443 No. (%)
Mean age (years) ± SD	74.1 ± 12.9	75.6 ± 11.6
Male, n = 684 (68.2)	369 (65.9)	315 (71.1)
Charlson comorbidity index		
Score over 1, n = 317 (31.6)	190 (33.9)	127 (28.7)
Antithrombotic treatment, n = 251 (25.0)	142 (25.4)	109 (24.6)
Vitamin K antagonist, n = 174 (17.3)	83 (14.8)	91 (20.5)
Preoperative GCS score*		
13–15, n = 911 (90.8)	503 (89.8)	408 (92.1)
9–12, n = 47 (4.7)	30 (5.4)	17 (3.8)
3–8, n = 24 (2.4)	14 (2.5)	10 (2.3)
CT hematoma density**		
Hyperdense, n = 215 (21.4)	134 (23.9)	81 (18.3)
Isodense, n = 429 (42.8)	257 (45.9)	172 (38.8)
Hypodense, n = 335 (33.4)	159 (28.4)	176 (39.7)
Mixed densities, n = 19 (1.9)	10 (1.8)	9 (2.0)

SD: Standard deviation

^*^21 patients with missing GCS values

^**^5 patients with missing characterization of CT hematoma density

Antithrombotic treatment was evenly represented in both treatment groups. Anticoagulation in the form of VKA was more present in the MC group (20.5% vs 14.8%, p = 0.020 Mann–Whitney). The preoperative GCS scores were similar in both groups with a median of 15 and with most scores between 13 and 15 in both treatment groups (89.8% and 92.1%).

Data concerning surgical characteristics and outcome was also collected for the study cohort (Table 2). The mean hematoma diameter for the study cohort was 22.5 mm with comparable diameters in both treatment groups. The midline shift was however found to be significantly larger in the MC group (p = 0.013). Most patients were operated in local anesthesia with light sedation (97% in the BHC group vs 88.3% in the MC group). The drainage systems were inserted for similar durations (1.0 in the BHC group vs 1.2 days in the MC group).

**Table 2:** Surgical characteristics and outcome data of the study population. The data in the following table is stratified for the two surgical methods

Variable No. (%)	BHC with active subgaleal drain N = 560 No. (%)	Minicraniotomy with passive subdural drain N = 443 No. (%)
Mean midline shift (mm) ± SD	8.8 ± 4.2	9.5 ± 4.2
Mean axial hematoma diameter (mm) ± SD	22.5 ± 6.4	22.4 ± 5.6
Local anesthesia with light sedation, n = 934 (93.1)	543 (97.0)	391 (88.3)
Mean time with drainage system (days) ± SD	1.0 ± 0.06	1.2 ± 0.44
Reoperated within 3 months after primary evacuation, n = 98 (9.8)	50 (8.9)	48 (10.8)
30-day mortality, n = 25 (2.5)	13 (2.3)	12 (2.7)
1-year mortality, n = 112 (11.2)	70 (12.5)	42 (9.5)
Complications according to Ibañez, n = 131 (13.1)	35 (6.3)	96 (21.7)
Grade Ia: complication requiring no drug treatment	5 (0.90)	21 (4.7)
Grade Ib: complication requiring drug treatment	17 (3.0)	50 (11.3)
Grade IIa: complication requiring intervention without general anesthesia	5 (0.9)	6 (1.4)
Grade IIb: complication requiring intervention with general anesthesia	1 (0.2)	10 (2.3)
Grade IIIa: complication involving single organ failure and ICU care	2 (0.4)	1 (0.20)
Grade IIIb: complication involving multiple organ failure and ICU care	0 (0.0)	0 (0.0)
Grade IV: complication resulting in death	5 (0.9)	8 (1.8)

SD: Standard deviation

### Recurrence rates in the surgical treatment models

The recurrence rates for the entire study population were 9.8% (n = 98). Recurrence rate was 10.8% (n = 48) for patients operated with MC and 8.9% (n = 50) for patients operated with BHC. The odds ratio for reoperation after treatment with MC was 1.24 and non-significant as compared to surgery with BHC (p = 0.336, 95% CI 0.82–1.88).

### Complication rates in the surgical treatment models

Complications were categorized by using the Landriel Ibañez system for complications. The study cohort had a total of 131 complications (13.1%) with 35 (6.3%) in patients who had undergone treatment with BHC. The remaining 96 complications (21.7%) were associated with MC (Table 2).

Complications were analyzed in regard to the two treatment groups with a statistically significant difference (p < 0.001). The analysis did however not give sufficient insight into the subgroups, and thus, post hoc testing was used yielding significant adjusted residual values (> 2) for Ibañez types Ia, Ib, and IIb (Table 3).

**Table 3:** Univariable analysis of complications associated with the two treatment groups. All tests were done with Fisher’s exact test

Complication type	BHC with active subgaleal drain N = 560 (%)	Minicraniotomy with passive subdural drain N = 443 (%)	P-value	CI 95%	Adjusted residual value
Grade Ia: complication requiring no drug treatment	5 (0.90)	21 (4.7)	0.001*	2.1–14.8	3.8
Grade Ib: complication requiring drug treatment	17 (3.0)	50 (11.3)	0.001*	2.3–7.2	5.2
Grade IIa: complication requiring intervention without general anesthesia	5 (0.9)	6 (1.4)	0.550	0.5–5.0	0.7
Grade IIb: complication requiring intervention with general anesthesia	1 (0.2)	10 (2.3)	0.003*	1.6–101.2	3.1
Grade IIIa: complication involving single organ failure and ICU care	2 (0.4)	1 (0.2)	1.000	0.1–7.0	− 0.4
Grade IV: complication resulting in death	5 (0.9)	8 (1.8)	0.263	0.7–6.3	1.3

^*^Significant p-value

The complication types were compared between the treatment groups with exception for Ibañez IIIb where no cases were recorded. We identified complications categorized as Ibañez Ia, Ib, and IIb to have a significantly increased odds ratio of being present in the treatment group of MC (Table 3). As was previously indicated to us by the abnormally high adjusted residual values.

Detailed analysis of Ibañez IIb complications in the MC group revealed that out of the 10 reported IIb complications, 8 had postoperative acute subdural hematomas, 1 postoperative epidural hematoma, and 1 subdural empyema (Table 4).

**Table 4:** Characterization of Ibañez IIb complications in the MC subgroup

Type of IIb complication	Total complications n = 10 (%)
Postoperative acute subdural hematoma	8 (80.0)
Postoperative epidural hematoma	1 (10.0)
Postoperative subdural empyema	1 (10.0)

### 30-day mortality rate in the surgical treatment models

**Fig. 1:**
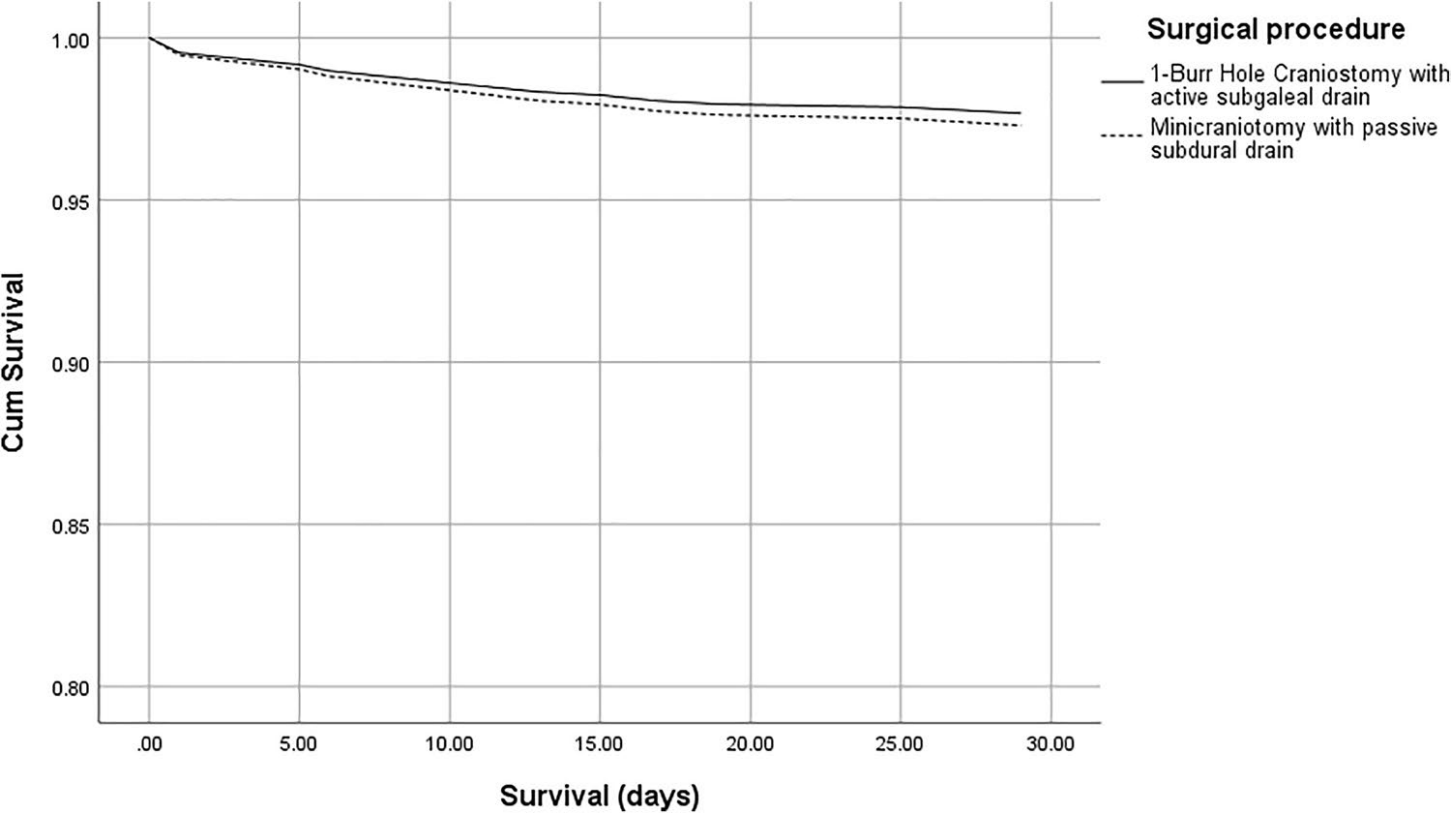
Survival function curve displaying the non-significant difference in 30-day mortality between the two treatment groups

Mortality was calculated from the time of surgery to the time of death. A 30-day and 1-year mortality was registered for all study patients. A 30-day mortality for the study cohort was found to be 2.5% (n = 25) with even distribution between the two treatment models (p = 0.696, Mann–Whitney). This was further tested in a survival function test. The hazard ratio for mortality at 30-day post-surgery with MC was 1.17 and non-significant as compared to BHC (p = 0.701, 95% CI 0.53–2.56, Cox regression) (Fig. 1).

## Discussion

In this retrospective cohort study involving patients with unilateral CSDH undergoing surgery with two different techniques, BHC with active subgaleal drain or MC with passive subdural drain, we found no significant differences in outcome concerning mortality or recurrence. However, there was a significant difference in reported complications favoring BHC with active subgaleal drain compared to MC with passive subdural drain.

The recurrence rate in our study was in line with previously reported recurrence rates for CSDHs. When comparing our two surgical methods, we were not able to identify a significant difference in recurrence rate. Previous studies have highlighted the benefits of a MC due to better visualization and access to the hematoma and membranes [27]. The MC has been theorized to be better suited for recurrent CSDHs but also to prevent recurrence in the first place. There are however other studies with no major differences in outcomes such as recurrence when comparing MC to BHC [16, 20]. There is also data supporting the use of only one burr hole instead of two burr holes with similar results [5, 23, 28]. Regarding drainage techniques, initial studies proved the efficacy of subdural drains [23], whereas subsequent studies on active subgaleal drains seem to be of similar effectiveness without the risk of misplacement in the brain parenchyma. [9, 12, 13, 24, 26].

In our study, there was no difference in mortality between the groups. The 1-year mortality rate in our study cohort was 11.2%, which is in line with previous studies [19, 30]. This contrasts the 1-year mortality rate for the general Swedish population between 75 and 79 years of age being approximately 3% [6]. We thus confirm that CSDH seems to be a sentinel health event [8, 14, 17].

In our study, we found a higher rate of complications associated with MC. The rate of Ia and Ib complications (medical complications) in the MC group in our study was 16%, which is in line with previous studies [21, 27]. MC was also associated with significantly more type IIb complications. Previous studies have found an increased rate of serious complications and morbidity when operating with a craniotomy [7, 16]. However, it is important to note that those studies are referring to a more invasive technique compared to MC. Van der Weken et al. specifically studied MC but could not verify that the technique was associated with serious surgical complications [27].

The study is inherently limited by the retrospective study design. In this study, we chose to include unilateral hematomas, which are reflected in the relatively low recurrence rates. Bilateral CSDH’s are associated with an increased risk of recurrence [2, 31]. The study is limited by the dyssynchronous time periods between the two centers, even though we have no clear indications that this would affect the results regarding recurrence or complications. The strength of the study is the relatively large study population and a health care system where no patients with CSDH are treated in private clinics, reducing the risk of selection bias. It also enables us to register complications, especially severe complications requiring surgery, since no surgical complications will be handled outside the neurosurgical departments at our hospitals.

## Conclusion

In this retrospective comparative cohort study, we found that the less invasive BHC technique holds at least equivalent effectiveness to MC. We also found that surgical evacuation by MC was significantly associated with medical complications as well as surgical complications, including postoperative acute subdural hematomas indicating a better safety profile when using the BHC technique.

## Abbreviations

CSDH: Chronic subdural hematoma
BHC: One burr hole craniostomy with active subgaleal drain
MC: Minicraniotomy with passive subdural drain
CCI: Charlson comorbidity index
